# Transarterial Radioembolisation with Y90 Resin Microspheres and the Effect of Reimbursement Criteria in France: Final Results of the CIRT-FR Prospective Observational Study

**DOI:** 10.1007/s00270-024-03955-y

**Published:** 2025-01-14

**Authors:** M. Ronot, R. Loffroy, D. Arnold, M. Greget, C. Sengel, J. B. Pinaquy, O. Pellerin, G. Maleux, B. Peynircioglu, J. P. Pelage, N. Schaefer, B. Sangro, N. de Jong, B. Zeka, M. Urdaniz, T. Helmberger, V. Vilgrain

**Affiliations:** 1 Department of Radiology, Hôpital Beaujon APHP Nord, Université Paris Cité, Paris, CRI, INSERM, 1149 Clichy, France; 2 Department of Vascular and Interventional Radiology, Image-Guided Therapy Center, CHU Dijon Bourgogne, François-Mitterrand University Hospital, 14 Rue Gaffarel, 21000 Dijon, France; 3 Oncology and Hematology, Asklepios Tumorzentrum Hamburg, AK Altona, Paul-Ehrlich-Str. 1, 22763 Hamburg, Germany; 4 Imagerie Interventionnelle UF 7524 Hôpital de Hautepierre, Hôpitaux Universitaires de Strasbourg, 67200 Strasbourg, France; 5 Interventional Radiology, Centre Hospitalier Universitaire de Grenoble, Boulevard de La Chantourne, 38100 Grenoble, France; 6 Department of Nuclear Medicine, CHU Bordeaux, 33000 Bordeaux, France; 7 Department of Vascular and Oncological Interventional Radiology, AP-HP, Hôpital Européen Georges Pompidou, HEKA INRIA, INSERM PARCC U 970, Université de Paris Cité, 20 Rue LEBLANC, 75015 Paris, France; 8 Radiology, Universitair Ziekenhuis Leuven, Herestraat 49, 3000 Louvain, Belgium; 9 Department of Radiology, School of Medicine, Hacettepe University, Sihhiye Campus, 06100 Ankara, Turkey; 10 Department of Diagnostic Radiology, McGill University Health Centre (MUHC – Glen) – Royal Victoria Hospital, Montreal, Canada; 11 Service de Médecine Nucléaire Et Imagerie Moléculaire, CHUV, Centre Hospitalier Universitaire Vaudois, Rue du Bugnon 46, 1011 Lausanne, Switzerland; 12 Liver Unit and HPB Oncology Area, Clínica Universidad de Navarra and CIBEREHD, Avda. Pio XII 36, 31008 Pamplona, Spain; 13 P+F Products and Features GmbH, Bösendorferstraße 5/3, 1010 Vienna, Austria; 14 Clinical Research Department, Cardiovascular and Interventional Radiological Society of Europe, Neutorgasse 9, 1010 Vienna, Austria; 15 Department of Radiology, Neuroradiology and Minimal-Invasive Therapy, Klinikum Bogenhausen, Englschalkinger Str. 77, 81925 Munich, Germany

**Keywords:** Hepatocellular carcinoma, Colorectal liver metastases, Transarterial radioembolisation, Yttrium-90, Resin microspheres, Liver, Reimbursement

## Abstract

**Purpose:**

This analysis of the CIRSE Registry for SIR-Spheres Therapy in France, CIRT-FR, reports on real-world outcomes of transarterial radioembolisation (TARE) with Y90 resin microspheres for hepatocellular carcinoma (HCC) and colorectal cancer liver metastases (CRLM) patients in France, focusing on safety, effectiveness and health-related quality of life (HRQoL). Results on patients treated based on national reimbursement criteria are discussed here.

**Methods:**

Prospective, multicentre, observational study of HCC and CRLM patients treated between August 2017 and July 2020 with TARE Y90 resin microspheres. Patients were assigned to different analysis groups based on reimbursement recommendations. Follow-up period was at least 24 months with patient data collected every 3 months.

**Results:**

In total, 252 (193 HCC, 59 CRLM) patients of CIRT-FR were included in the analysis. No differences in effectiveness, safety and HRQoL were found between analysis groups based on reimbursement recommendations. Median overall survival for HCC and CRLM was 19.0 (95% CI, 16.1–22.4) and 10.8 (95% CI, 8.0–13.5) months, respectively. Serious procedure-related adverse events occurred in 13% of the patients. HRQoL generally remained stable, with some fluctuations in function scores and symptoms.

**Conclusion:**

In our cohorts, patients performed similarly regarding clinical outcomes irrespective of their analysis group based on reimbursement recommendations. Our results suggest that instead of restrictive reimbursement criteria, more decision-making power in selecting suitable patient groups could be given to multidisciplinary tumour boards. Results confirm that TARE with Y90 resin microspheres is an effective and safe treatment for liver cancer, with maintained HRQoL in most patients.

**Graphical abstract:**

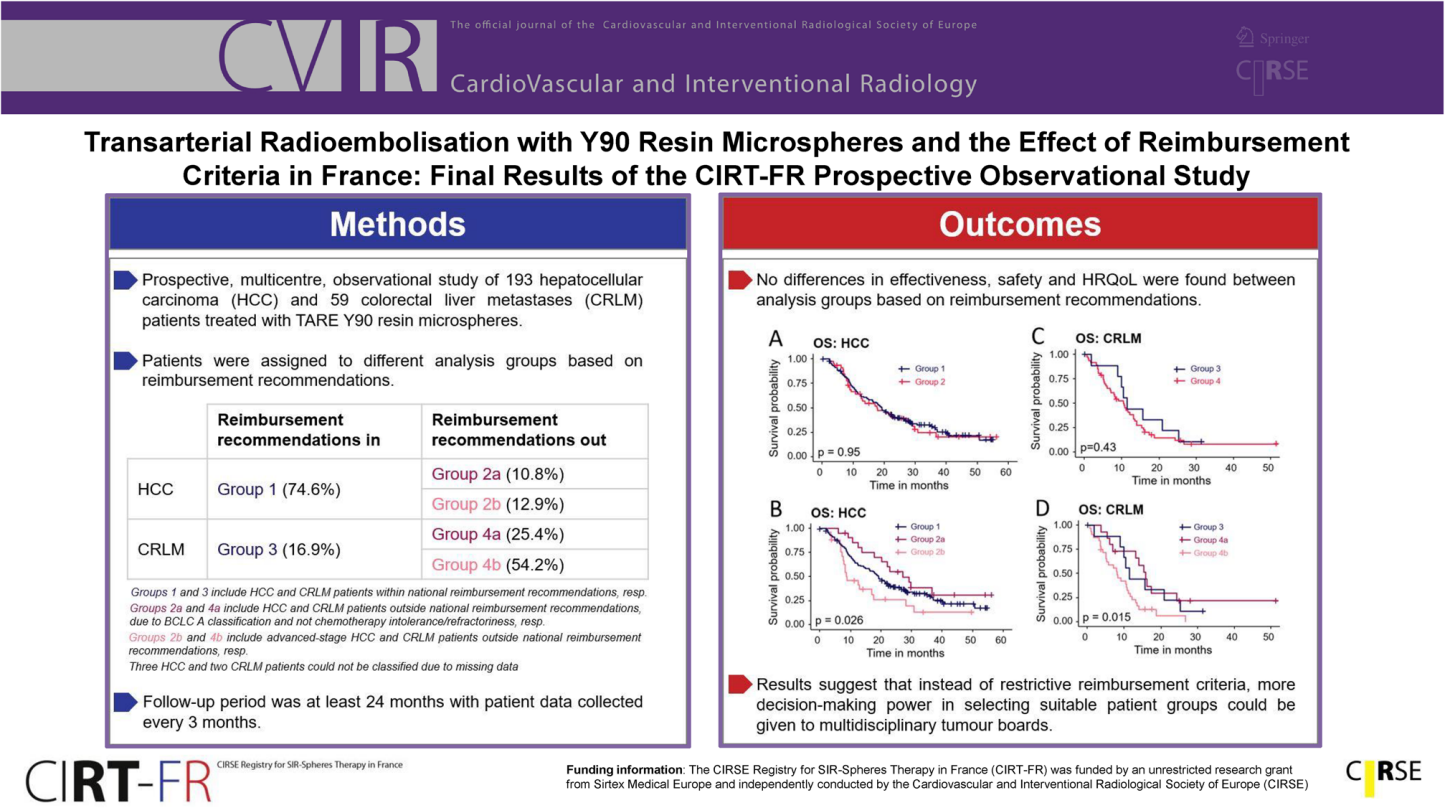

**Supplementary Information:**

The online version contains supplementary material available at 10.1007/s00270-024-03955-y.

## Introduction

Transarterial radioembolisation (TARE) is a treatment option for patients with unresectable primary and secondary metastatic liver cancer and is thus considered a viable therapeutic approach for hepatocellular carcinoma (HCC), and colorectal liver metastases (CRLM) in several treatment guidelines and recommendations [1–4].

Recent advancements in the application of TARE in dosimetry calculation and precision of dose delivery showed improved effectiveness in terms of reported overall survival [5, 6], with similar rates of toxicity compared to less accurate dosimetry models [7, 8]. Further, explorations on the biological effect of TARE on local and systemic immune responses show promising results concerning potential combination therapies of TARE with systemic treatment modalities, including immunotherapy and targeted therapies [9–12]. At the same time, real-world cohort studies gained valuable insights into the real-world effectiveness and safety outcomes of TARE [8, 13–21], showing that it has a strong evidence base in unresectable liver tumours and can even have a place as a downstaging procedure to improve access to resection of metastases, and/or liver transplantation [6, 22–27].

Given the position of TARE in guidelines at the time and the growing interest by regulatory and reimbursement agencies in real-world evidence to complement data from more controlled settings [28–31], the French national health authorities (Haute Autorité de Santé [HAS]) granted the reimbursement of Y90 resin microspheres for selected CRLM and HCC patients, based on predefined criteria related to disease and patient status in 2015 [32] and 2018 [33], respectively. Conditional for reimbursement was the collection of real-world evidence in France.

The Cardiovascular and Interventional Radiological Society of Europe (CIRSE) therefore initiated the CIRSE Registry for SIR-Spheres Therapy in France (CIRT-FR) [20], as an offshoot of the European-wide CIRSE Registry for SIR-Spheres Therapy (CIRT) [17]. The purpose of the study was to collect prospective data to aid the assessment of the safety and effectiveness of Y90 resin microspheres (SIR-Spheres®, Sirtex Medical Limited, St. Leonards, NSW, Australia) by the HAS, which uses real-world data to inform their reimbursement policies. Here, the authors report on the real-life clinical application of Y90 resin microspheres in France, the applicability of the HAS recommendations for reimbursement, and safety and effectiveness outcomes, including health-related quality of life (HRQoL).

## Methods

### Study Design

CIRT-FR is a prospective, multicentre, single-device observational study of patients with primary and secondary metastatic liver cancer treated with TARE with Y90 resin microspheres, entirely recruited in France. Since CIRT-FR was an offshoot of CIRT, it adopted its methodology, which has been published previously [34].

### Site Selection and Patient Inclusion

The study aimed to enrol all patients treated with TARE with Y90 resin microspheres. Therefore, sites were invited to participate if they had treated at least one patient with Y90 resin microspheres in the past or if they had expected to perform their first TARE in the near future. From April 2017 to June 2020, 26 sites were initiated, of which 14 included patients.

Inclusion criteria were adult patients scheduled to be treated with TARE with Y90 resin microspheres. There were no specific exclusion criteria as the study aimed for broad patient coverage. Recruitment occurred between 1 August 2017 and 31 July 2020. Follow-up data were collected until 31 July 2022; patients were followed up for at least 24 months or until study exit. It was recommended that follow-up data be collected every 3 months, but exact follow-up intervals were left to the discretion of the medical teams. In case follow-up evaluations were not performed at the site of the TARE treatment, sites were encouraged to obtain follow-up information from referring physicians.

All procedures performed were in accordance with the ethical standards of the national ethics committee (N° ID-RCB: 2017-A01003-50) and with the 1964 Helsinki Declaration and its later amendments or comparable ethical standards. All patients signed an informed consent form.

### HAS Recommendations for Reimbursement

Y90 resin microspheres received reimbursement approval from the HAS for CRLM and HCC patients in 2015 and 2018, respectively.

#### Criteria for Reimbursement in CRLM Patients

- Preserved general status, ECOG^1^ performance status (PS) ≤ 2
- Absence of significant liver tumour invasion, tumour burden < 25%
- Absence of extrahepatic disease
- Refractory or intolerant to all recognised intravascular (IV) and oral therapies

#### Criteria for Reimbursement in HCC Patients

- Intermediate or advanced HCC, BCLC^2^ B/C
- Absence of complete portal vein thrombosis (PVT)
- Preserved general status, ECOG PS < 2
- Preserved liver function (Child–Pugh A or B)
- Not eligible or failed sorafenib

### Study Objectives

The primary objective was to observe the real-world application of TARE with Y90 resin microspheres in the context of national criteria for reimbursement. Since reimbursement criteria could play a critical role in the accessibility of TARE with Y90 resin microspheres to patients, the influence of the reimbursement criteria set by the HAS on patient selection and treatment proceedings was evaluated.

Subgroups of patients based on reimbursement recommendations were assessed as follows:

### HCC

- *Group 1 (within reimbursement criteria)* Intermediate- or advanced-stage HCC patients (BCLC B/C), absence of complete PVT, ECOG < 2, Child–Pugh A or B and not eligible or failed to sorafenib
- *Group 2 (outside reimbursement criteria)*
  - *Group 2a* BCLC A HCC patients
  - *Group 2b* HCC patients with at least one of the following: complete PVT, ECOG ≥ 2, Child–Pugh C

### CRLM

- *Group 3 (within reimbursement criteria)* CRLM patients with all the following: ECOG ≤ 2, tumour burden < 25%, absence of extrahepatic disease and refractory or intolerant to all recognised IV and oral therapies
- *Group 4 (outside reimbursement criteria)*
  - *Group 4a* Not chemotherapy–refractory/intolerant CRLM patients
  - *Group 4b* CRLM patients with at least one of the following: ECOG ≥ 3, tumour burden ≥ 25%, presence of extrahepatic disease

Secondary objectives include effectiveness, determined by overall survival (OS), progression-free survival (PFS), hepatic PFS (hPFS), safety and the impact of the treatment on the HRQoL of the patient.

### Assessments

At the time of the first treatment, baseline demographics and treatment-related data were collected. Eastern Cooperative Oncology Group (ECOG) and Barcelona Clinic Liver Cancer (BCLC) staging were calculated at baseline, the latter following the updated algorithm [3] based on data provided by the centres. Information concerning post-TARE treatments, safety and time-to-event data was gathered at every follow-up visit. Time-to-event outcome measures were assessed from the day of TARE treatment until the date of the event. Liver function was described using the albumin–bilirubin (ALBI) formula developed by Johnson et al.: ALBI score = (log10 bilirubin [μmol/L] × 0.66) + (albumin [g/L] ×  − 0.0852). ALBI score ≤  − 2.60 is grade 1, >  − 2.60 to ≤  − 1.39 is grade 2, and >  − 1.39 is grade 3 [35]. Safety outcomes are described according to the Common Terminology Criteria for Adverse Events, version 4.03. Pre-defined serious adverse events (grade 3 and 4) were abdominal pain, fatigue, fever, nausea, vomiting, gastrointestinal ulceration, gastritis, radiation cholecystitis, radiation pancreatitis and radioembolisation-induced liver disease (REILD). An open text field allowed for collecting details on other serious adverse events. HRQoL was collected at baseline, within one week after treatment, and at every follow-up using the EORTC QLQ-C30 [36, 37] and the HCC [38] module.

### Statistical Analysis

Data are presented as mean ± SD or median (IQR) for continuous variables and number (%) for categorical variables. Percentages are based on the indication-specific cohorts unless otherwise indicated.

OS, PFS and hPFS were calculated with the associated 95% confidence interval using the Kaplan–Meier method. Patients who died during the study were categorised as having progression for the purpose of PFS and hPFS analysis. Patients alive and progression-free were censored on the day of the last follow-up. Comparisons of OS were performed using the log-rank test and a *p*-value of < 0.05 was considered statistically significant. Multivariable survival analysis for OS, PFS and hPFS was performed using a Cox proportional hazards model. The selection of variables was determined following a univariable analysis and a subsequent stepwise variable selection procedure with a significance level of 0.05 when deciding to enter a predictor into the stepwise model.

For the final model, the model with the lowest Akaike information criterion value was used. To calculate statistical differences between the frequency of AEs, the Chi-squared test was used. To determine improved or deteriorated HRQoL, a change of 10 items points was used as a cut-off. Two-sided one-sample paired t-tests (or Wilcoxon signed-rank tests) were employed to test statistical significance of changes of follow-up HRQoL from baseline HRQoL. Shapiro–Wilk test was used to test the normality assumptions on changes from baseline. All available data were used, and no imputations of missing data were made. All statistical analyses were performed using R Studio under R.

## Results

**Fig. 1:**
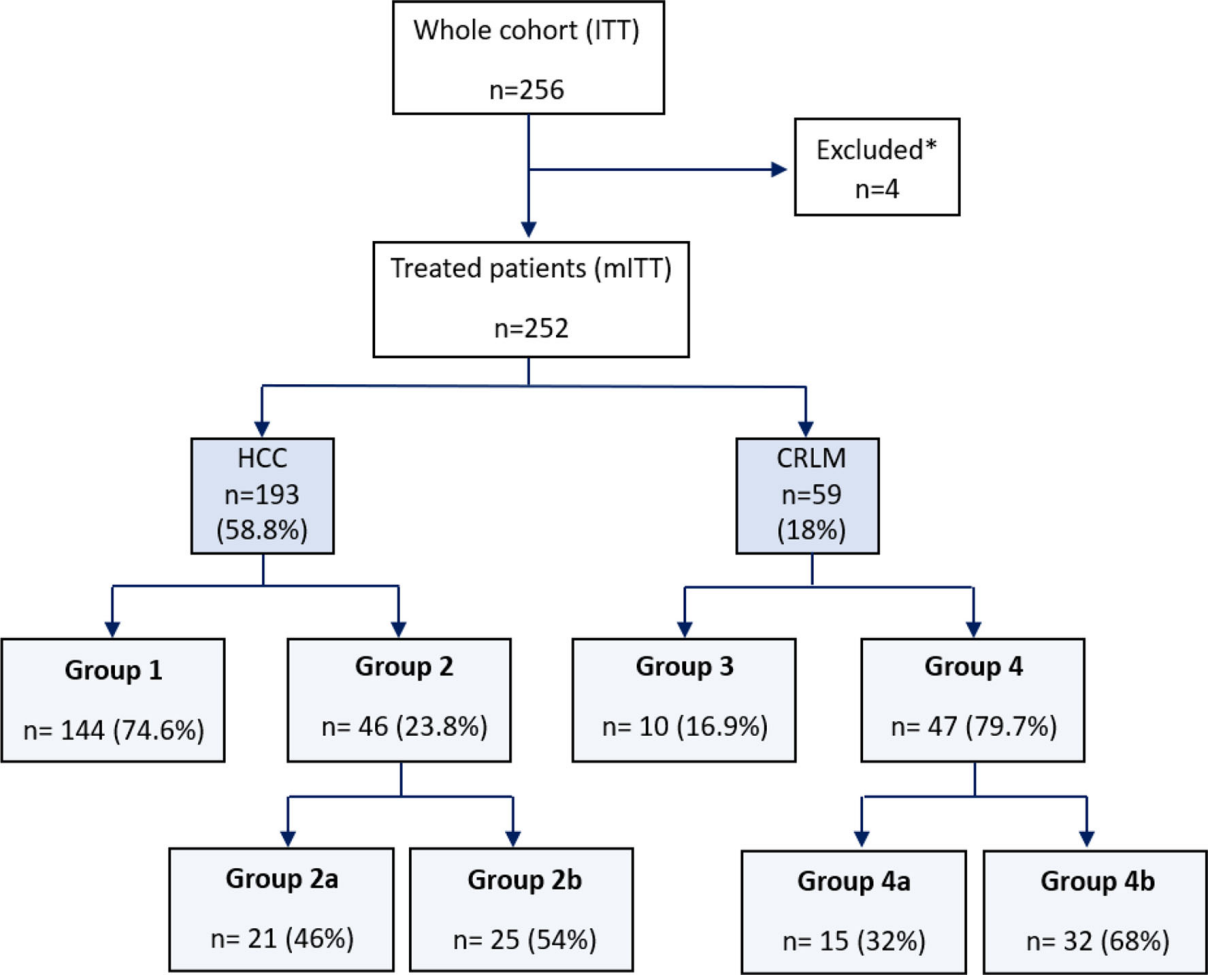
Patient allocation by indication and by analysis group based on HAS reimbursement recommendations. *Reasons for patient exclusion were: treatment cancelled due to lack of tumour uptake of MAA during workup (*n* = 1), due to coil migration during workup (*n* = 1) and contraindication to perform hepatic radioembolisation due to a defect of arterialisation of secondary lesions (*n* = 1). For one patient no reason was provided. Three HCC and two CRLM patients could not be classified into analysis groups due to missing data. Abbreviations. (m)ITT: (modified) intention-to-treat; HAS: Haute Autorité de Santé; MAA: macroaggregated albumin; HCC: hepatocellular carcinoma; CRLM: colorectal liver metastases

A total of 256 HCC and CRLM patients were enrolled in the study, of which four were excluded (see Fig. 1). Data from 193 (58.8%) HCC and 59 (18%) CRLM patients were included in the analysis. The median age was 68 (IQR 61.0–75.0) and 64 (IQR 55.5–70.0) years for the HCC and CRLM cohorts, respectively; 201 (79.8%) patients were male (Table 1). More information on baseline and treatment is given in Table 1 and Supplementary Table 1.

**Table 1:** Baseline characteristics

	HCC	CRLM
*Total N (%)*		
	193 (58.8)	59 (18.0)
*Analysis groups*		
Group 1 (HCC); Group 3 (CRLM)	144 (74.6)	10 (16.9)
Group 2 (HCC); Group 4 (CRLM)	46 (23.8)	47 (79.7)
Cannot be determined	3 (1.6)	2 (3.4)
*Age*		
Median (IQR)	68.0 (61.0–75.0)	64.0 (55.5–70.0)
*Sex*		
Female	25 (13.0)	26 (44.1)
Male	168 (87.0)	33 (55.9)
*ECOG performance status*		
0	85 (44.0)	22 (37.3)
1	92 (47.7)	26 (44.1)
2	11 (5.7)	4 (6.8)
3	1 (0.5)	1 (1.7)
(Missing)	4 (2.1)	6 (10.2)
*BCLC*		
A	21 (10.9)	–
B	27 (14.0)	–
C	136 (70.5)	–
D	8 (4.1)	–
(Missing)	1 (0.5)	–
*Child–Pugh Class*		
A (5–6)	133 (68.9)	–
B (7–8)	31 (16.1)	–
C (9 +)	7 (3.6)	–
Unknown	22 (11.4)	–
*Cirrhosis*		
No	49 (25.4)	59 (100.0)
Yes	144 (74.6)	0 (0.0)
*Ascites*		
No	187 (96.9)	59 (100.0)
Yes	6 (3.1)	0 (0.0)
*Extrahepatic disease*		
No	159 (82.4)	37 (62.7)
Yes	34 (17.6)	22 (37.3)
*Portal vein thrombosis status*		
Segmental thrombosis	52 (26.9)	6 (10.2)
Lobar thrombosis	17 (8.8)	1 (1.7)
Main thrombosis	7 (3.6)	3 (5.1)
Patent	117 (60.6)	49 (83.1)
*Prior procedures in the liver*		
Surgical procedures	20 (10.4)	16 (27.1)
Ablative procedures	30 (15.5)	10 (16.9)
Vascular procedures	90 (46.6)	11 (18.6)
Abdominal radiotherapy	3 (1.6)	2 (3.4)
No prior procedures	88 (45.6)	34 (57.6)
*Prior systemic chemotherapy lines*		
1	13 (6.7)	8 (13.6)
2	4 (2.1)	27 (45.8)
> 2	1 (0.5)	22 (37.3)
No prior systemic therapy	175 (90.7)	2 (3.3)
*Chemo-refractory or intolerant*		
No	183 (94.8)	30 (50.8)
Yes	10 (5.2)	29 (49.2)
*Liver tumour location*		
Bilobar	49 (25.4)	36 (61.0)
Left	41 (21.2)	5 (8.5)
Right	103 (53.4)	18 (30.5)
*Number of tumours*		
1	89 (46.1)	9 (15.3)
2–5	73 (37.8)	20 (34)
> 6	12 (6.2)	10 (17)
Uncountable	18 (9.3)	18 (30.5)
Unknown	1 (0.5)	2 (3.4)
*Hepatic tumour burden*		
≤ 25%	146 (75.6)	39 (66.1)
> 25%	37 (19.2)	15 (25.4)
(Missing)	10 (5.2)	5 (8.5)
*Lung shunt (%)*		
0 to 10%	187 (96.9)	59 (100.0)
> 10% to 15%	1 (0.5)	0 (0.0)
> 15% to 20%	3 (1.6)	0 (0.0)
> 20%	1 (0.5)	0 (0.0)
Unknown	1 (0.5)	0 (0.0)
*Method for determining dose*		
BSA	25 (13.0)	25 (42.4)
Empiric	0 (0.0)	1 (1.7)
Modified BSA	28 (14.5)	4 (6.8)
Other	1 (0.5)	4 (6.8)
Partition model	139 (72.0)	24 (40.7)
Unknown	0 (0.0)	1 (1.7)
*Concomitant chemotherapy*		
No	185 (95.9)	46 (78.0)
Yes	8 (4.1)	13 (22.0)
*ALBI grade*		
1	23 (11.9)	15 (25.4)
2	127 (65.8)	24 (40.7)
3	10 (5.2)	4 (6.8)
(Missing)	33 (17.1)	16 (27.1)
*Intent of SIR-Spheres therapy*		
Ablation (radiation segmentectomy)	15 (7.8)	4 (6.8)
Bridge to liver transplant	19 (9.8)	2 (3.4)
Downsizing/downstaging	15 (7.8)	11 (18.6)
Palliative (cytoreduction)	144 (74.6)	42 (71.2)

*HCC* hepatocellular carcinoma, *CRLM* colorectal liver metastases, *IQR* interquartile range, *ECOG* Eastern Cooperative Oncology Group, *BCLC* Barcelona Clinic Liver Cancer, *(m)BSA* (modified) body surface area, *ALBI* albumin–bilirubin

### HCC

In the HCC cohort (193, 58.8%), most patients had ECOG 0 (44.0%) or 1 (47.7%) and BCLC C (70.5%). Child–Pugh was mainly A (68.9%). Most of the patients had cirrhosis (74.6%) at the time of inclusion, and a minority (3.1%) presented with ascites. There was no evidence of any extrahepatic disease in 82.4% of the patients, and the tumoural occlusion of the main portal vein was reported in 3.6%. Hepatic tumour burden was ≤ 25% in 75.6% of the patients. 54.4% of the patients had received any previous liver-directed procedures, and 9.3% had received systemic chemotherapy. The methods to determining the radiation dose were partition model (72%) and body surface area (BSA) or modified BSA (27.5%) (Table 1).

**Fig. 2:**
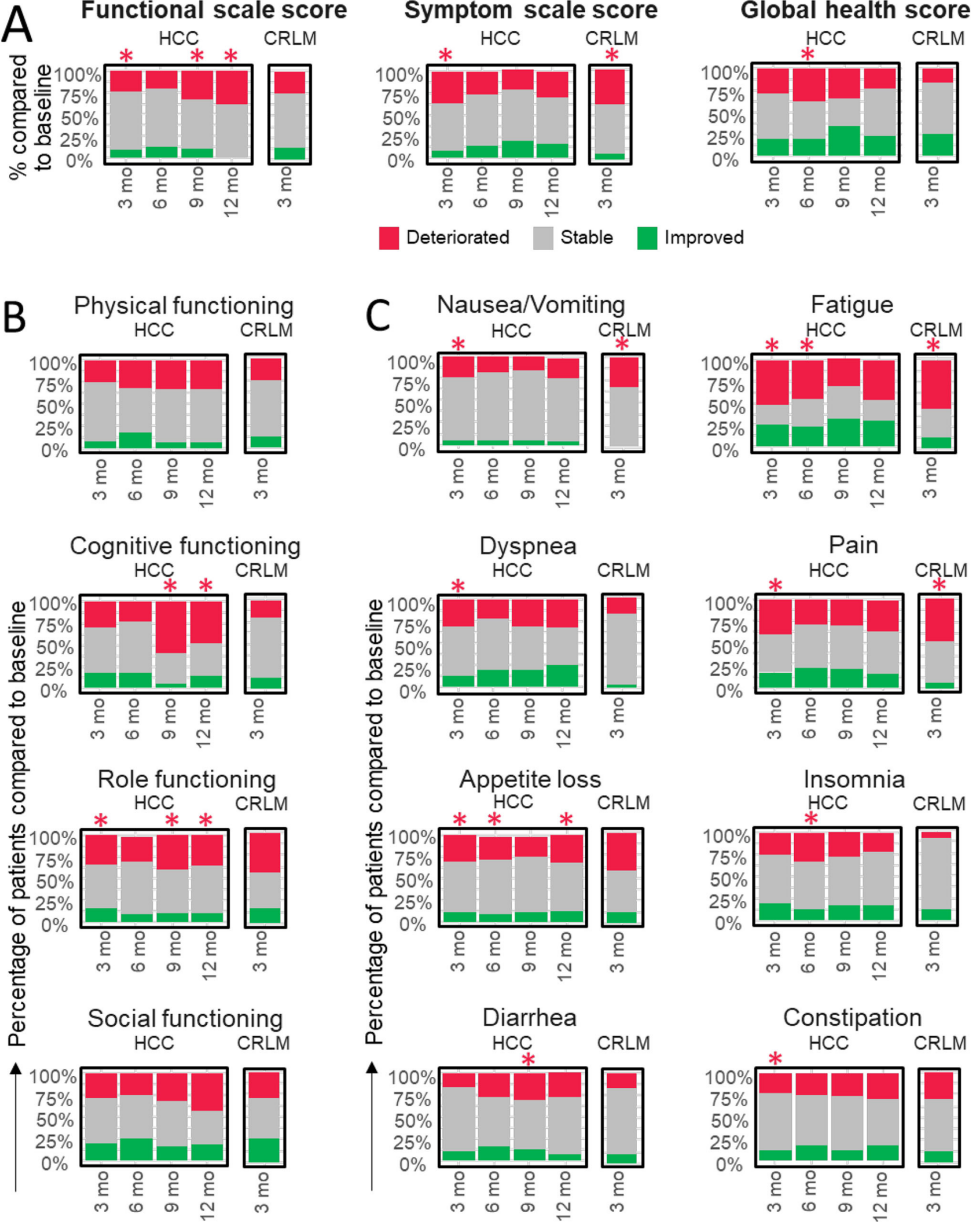
Health-related quality of life (HRQoL) changes over time. The figure illustrates the change in HRQoL measured using the EORTC QLQ-C30 for HCC at 3 (*n* = 91), 6 (*n* = 48), 9 (*n* = 41) and 12 months (*n* = 29), and CRLM at 3 months (*n* = 25). Proportions of patients with changes larger than 10 points worse than baseline, larger than 10 points better or within this range are depicted in red, green and grey, respectively. Two-sided one-sample paired t-test p-values (or Wilcoxon signed-rank test *p*-values) were reported for the statistical significance of changes from baseline. Shapiro–Wilk test was used to test the normality assumptions on changes from baseline. A red star indicates a statistically significant (*p* < 0.05) worsening compared to baseline. **A** General scores encompassing functional, symptom and global health assessments. **B** A detailed breakdown of the functional scores, including physical, cognitive, role and social functioning. **C** A detailed breakdown of the symptom scores nausea/vomiting, fatigue, dyspnoea, pain, appetite loss, insomnia, diarrhoea and constipation. Abbreviations. HCC: hepatocellular carcinoma; CRLM: colorectal liver metastases; EORTC QLQ: European Organization for Research and Treatment of Cancer Quality of Life Questionnaire

The median OS was 19.0 months (95% CI 16.1–22.4) with an interquartile range (IQR) of 8.6–28.4 months. Prognostic factors associated with shorter survival were hepatic tumour burden of > 25% (HR 1.7, 95% CI 1.1–2.7) and Child–Pugh B or C (HR 1.8, 95% CI 1.2–2.9). The partition model dosimetry method (HR 0.6, 95% CI 0.4–0.9) and post-TARE downstaging (HR 0.2, 95% CI 0.1–0.5)—defined as post-TARE ablation, resection or transplantation (36, 10.9%)—were associated with longer survival (Table 2). Prognostic factors for PFS and hPFS are shown in Supplementary Table 2. Overall, 76 (39.4%) HCC patients had at least one AE related to the procedure, of which 21 (27.6%) were of grade 3 or 4, most commonly fatigue (Supplementary Table 3). Regarding HRQoL assessment, significant deterioration was found in function scores at 3, 9 and 12 months and in global health at month 6. Symptoms significantly worsened at month 3. Global health status was maintained or improved in 73%, 66% and 76% of HCC patients at months 3, 9 and 12, respectively (Fig. 2).

**Table 2:** Effectiveness by indication

		Median OS (months, 95% CI)	Events	Censored	HR (95% CI)	*P*-value
*HCC*						
Whole cohort		19.0 (16.1–22.4)	134 (69.4)	59 (30.6)	–	–
*Analysis groups*						
Group 1		19.1 (14.9–22.7)	100 (69.4)	44 (30.6)	–	–
Group 2a		27.2 (17.7–NA)	13 (61.9)	8 (38.1)	–	–
Group 2b		8.9 (7.3–17.7)	19 (76.0)	6 (24.0)	–	–
*Tumour burden*						
≤ 25%		19.3 (14.9–26.6)	99 (67.8)	47 (32.2)	–	–
> 25%		12.6 (8.5–18.7)	32 (86.5)	5 (13.5)	1.7 (1.1–2.7)	0.028
*Child–Pugh Class*						
A (5–6)		22.1 (18.0–28.1)	85 (63.9)	48 (36.1)	–	–
B (7–8) or C (9 +)		9.6 (7.2–17.1)	32 (84.2)	6 (15.8)	1.8 (1.2–2.9)	0.007
*Dose methodology*						
BSA/mBSA		11.9 (8.9–19.2)	43 (79.6)	11 (20.4)	–	–
Partition model		21.2 (17.7–28.4)	91 (65.5)	48 (34.5)	0.6 (0.4–0.9)	0.025
*Post-TARE downstaging*						
No		17.9 (14.3–21.0)	123 (74.5)	42 (25.5)	–	–
Yes		Not reached	5 (23.8)	16 (76.2)	0.2 (0.1–0.5)	< 0.001
*CRLM*						
Whole cohort		10.8 (8.0–13.5)	50 (84.7)	9 (15.3)	–	–
*Analysis groups*						
Group 3		11.4 (1.8–25.3)	8 (80.0)	2 (20.0)	–	–
Group 4a		15.8 (6.1–24.4)	11 (73.3)	4 (26.7)	–	–
Group 4b		8.0 (5.0–10.8)	29 (90.6)	3 (9.4)	–	–
*Extrahepatic disease*						
No		13.1 (8.0–16.3)	31 (83.8)	6 (16.2)	–	–
Yes		9.0 (3.5–11.6)	19 (86.4)	3 (13.6)	4.0 (1.2–13.5)	0.023
*Number of tumours*						
1–5		15.4 (10.4–25.3)	21 (72.4)	8 (27.6)	–	–
> 5		10.2 (0.7–14.0)	10 (100)	0 (0)	2.8 (0.8–9.5)	0.094
*Bilobar disease*						
No		20.5 (10.6–26.6)	16 (69.6)	7 (30.4)	–	–
Yes		8.2 (5.4–11.2)	34 (94.4)	2 (5.6)	4.0 (1.3–12.5)	0.017
*ALBI score*						
1		13.5 (5.2–16.3)	15 (100)	0 (0)	–	–
3		1.3 (0.7–3.5)	4 (100)	0 (0)	147.7 (8.5–2566.2)	0.001

*OS* overall survival *CI* confidence interval *HR* hazard ratio *(m)BSA* (modified) body surface area *BCLC* Barcelona Clinic Liver Cancer *HCC* hepatocellular carcinoma *CRLM* colorectal liver metastases

### CRLM

In patients with CRLM (59, 18%), ECOG 0 and 1 were reported in 37.3% and 44.1% of the patients, respectively. No CRLM patient had cirrhosis or ascites at the time of inclusion. Extrahepatic disease was more prevalent than in other cohorts (37.3%). Main portal vein occlusion was reported in 5.1% of the patients. Almost half of the patients (42.4%) had received previous liver-directed procedures, whereas the majority (96.7%) had received prior systemic therapies. Of note, 49.2% of the patients were reported to be chemo-refractory or intolerant at the time of TARE. Hepatic tumour burden was > 25% in 25.4% of the patients, and most patients (61.0%) had bilobar tumours (Table 1).

The median OS was 10.8 months (95% CI 8.0–13.5) with an IQR of 5.1–16.0 months. Prognostic factors associated with shorter survival were extrahepatic disease (HR 4.0, 95% CI 1.2–13.5), more than 5 liver tumours (HR 2.8, CI 95% 0.8–9.5), bilobar disease (HR 4.0, 95% CI 1.3–12.5) and ALBI score 3 (HR 147.7, 95% CI 8.5–2566.2) (Table 2). Prognostic factors for PFS and hPFS are shown in Supplementary Table 2. Overall, 6 (10.2%) CRLM patients reported at least one procedure-related AE, mostly mild (4, 66.7%) (Supplementary Table 3). For HRQoL, significant increases in symptoms such as nausea or vomiting, pain and fatigue were found at month 3. Other than symptoms, global health status was generally maintained (60%) or improved (24%) in this cohort (Fig. 2).

### HCC and CRLM Patient Allocation into Analysis Groups

Overall, 23.8% of HCC and 79.7% of CRLM patients treated with TARE using Y90 resin microspheres were included in Group 2 and Group 4, respectively (see groups in the methods). Aside from data concerning the reimbursement criteria set by the HAS, no significant differences at baseline were found between Group 1 vs Group 2 and Group 3 vs Group 4 (Supplementary Tables 4A and 4B). Due to missing data, three HCC and two CRLM patients could not be allocated into the analysis groups (Fig. 1).

**Fig. 3:**
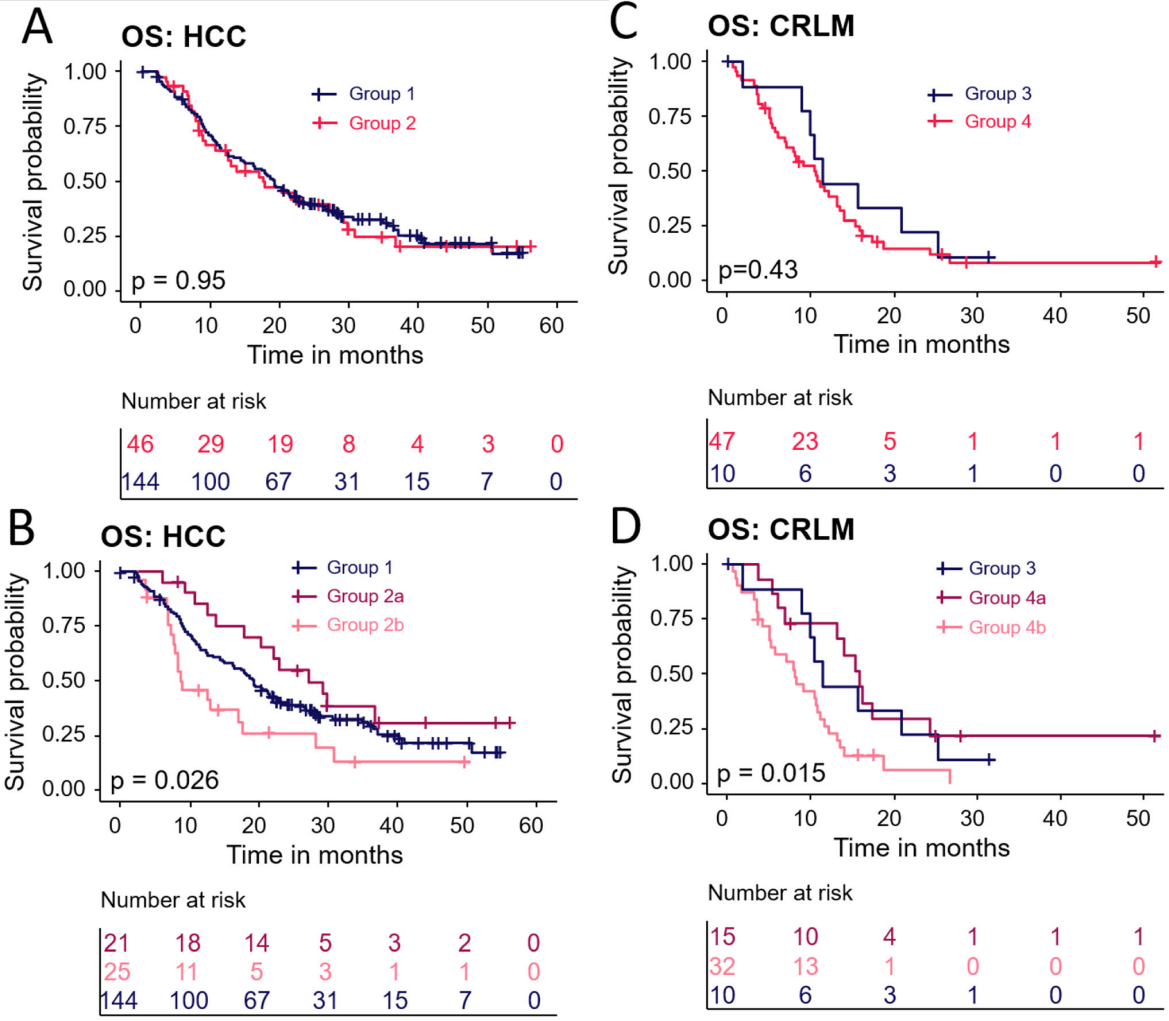
Overall survival (OS) for patients based on analysis groups. The figure shows OS plots using the Kaplan Meier method. Statistically significant differences between groups were assessed using the log-rank test. **A** OS of Group 1 (*n* = 144) and Group 2 (*n* = 46) HCC patients. **B** OS of Group 1 (*n* = 144), Group 2a (*n* = 21) and Group 2b (*n* = 25) HCC patients. *P*-values are as follows: Group 2b *vs* Group 1 *p* = 0.034, Group 2b *vs* Group 2a *p* = 0.001, Group 1 *vs* Group 2a *p* = 0.19. **C** OS of Group 3 (*n* = 10) and Group 4 (*n* = 47) CRLM patients. **D** OS of Group 3 (*n* = 10), Group 4a (*n* = 15) and Group 4b (*n* = 32) CRLM patients. P-values are as follows: Group 4b *vs* Group 3 *p* = 0.097, Group 4b *vs* Group 4a *p* = 0.008, Group 3 *vs* Group 4a *p* = 0.6. Abbreviations. HCC: hepatocellular carcinoma; CRLM: colorectal liver metastases

No differences in OS were found between main groups for either HCC (*p* = 0.95) or CRLM (*p* = 0.43, Fig. 3A and C). When subdividing the groups, patients from Group 2a, and therefore early-stage patients, had a significantly longer OS than those from Group 2b (*p* = 0.026, Fig. 3B). A similar situation was observed in the CRLM group, where patients included in Group 4a had longer survival than those from Group 4b (*p* = 0.015, Fig. 3D).

Regarding safety, no significant differences between any of the groups were found (see Table 3). Likewise, HRQoL assessment performed similarly in all groups. For HCC, significant differences were found in physical function at month 3 (*p* = 0.016), in social function score at month 12 (*p* = 0.007), in pain at month 3 (*p* = 0.027) and in constipation at month 9 (*p* = 0.027) following TARE (Supplementary Tables 5A and B). No significant differences in global health score were found (Supplementary Table 5C).

**Table 3:** Safety by indication and by analysis group

HCC: number of patients with at least 1 AE				
	Group 1	Group 2a	Group 2b	*P*–value
	*n* = 144	*n* = 21	*n* = 25	
**Any severity**				
*Related and unrelated*				
	102 (70.8)	14 (66.7)	21 (84)	0.34
*Related*				
	58 (40.3)	5 (23.8)	10 (40)	0.34
**Grade 3–4**				
*Related and unrelated*				
	38 (26.4)	6 (28.6)	8 (32)	0.84
*Related*				
	17 (11.8)	2 (9.5)	1 (4)	0.50

CRLM: number of patients with at least 1 AE				
	Group 3	Group 4a	Group 4b	*P*-value
	*n* = 10	*n* = 30	*n* = 17	
**Any severity**				
*Related and unrelated*				
	5 (50)	14 (46.7)	11 (64.7)	0.48
*Related*				
	1 (10)	3 (10)	2 (11.8)	0.98
**Grade 3–4**				
*Related and unrelated*				
	2 (20)	5 (16.7)	8 (47.1)	0.07
*Related*				
	0 (0)	5 (16.7)	8 (47.1)	0.30

*AE* adverse event *BCLC* Barcelona Clinic Liver Cancer *HCC* hepatocellular carcinoma *CRLM* colorectal liver metastases

## Discussion

CIRT-FR was conducted to aid the HAS in evaluating the reimbursement of Y90 resin microspheres for HCC and CRLM patients in France. Our data showed that clinical outcomes of TARE with Y90 resin microspheres perform similarly in patients from Group 1 when compared to patients from Group 2, as well as in patients from Group 3 when compared to patients from Group 4. When further subdividing the groups, it was noted that these similarities could be attributed to the better performance of fitter patients from Group 2a and Group 4a when compared to more advanced-stage patients from Group 2b and Group 4b.

Data from this study showed that 23.8% of the HCC and 79.7% of the CRLM patients were included in Groups 2 and 4, indicating that multidisciplinary tumour board’s (MDB) decision on TARE treatments is an important factor for treatment selection. Considering that the difference in survival and safety outcomes between groups was not statistically significant, a wider group of patients with HCC or CRLM might benefit from treatment with TARE with Y90 microspheres than initially recognised.

For CRLM patients, the decision in 2015 to reimburse Y90 resin microspheres was based on the assessment of five retrospective studies, one prospective and two systematic reviews conducted between 2008 and 2015 [32, 39–46] after previous assessments in 2009 and 2010 performed with insufficient data to warrant the reimbursement of Y90 resin microspheres. After 2015, several prospective and retrospective registries with consecutive patient enrolment were conducted in Europe and the USA, including the present study [15, 17, 47, 48]. The assessment of these studies led to an extension of the reimbursement criteria in 2022 and a more prominent role for MDB in the decision-making process. Moreover, the refractoriness criterion was modified from “refractory to all recognised intravascular and oral therapies” to “refractory or intolerant to all recommended systemic therapies” [49]. The nuanced addition of *recommended* systemic therapies to the criteria connects well with our survival findings, where we observed patients considered outside of the recommendations, as they were not chemotherapy–refractory or intolerant (Group 4a), displaying longer survival rates than patients outside of the recommendations due to other reasons (Group 4b) (*p* = 0.015, Fig. 3D). Likewise, these findings align with those of Emmons and colleagues, who observed that the longest OS was achieved when TARE was administrated as second line treatment in their prospective cohort [15].

In 2018, TARE became reimbursed for HCC patients with BCLC B or C without complete occlusion of the main portal vein, ECOG 0 or 1 with preserved liver function who were not eligible for or failed sorafenib [33]. This was based on two randomised controlled trials [50, 51] and three retrospective studies [52–54]. Based on recently updated guidelines and recent studies [7, 13, 17, 50, 55–57], including the present study, these criteria were extended in 2022 to include patients for whom the MDB decided not to provide TACE or systemic treatment [49]. Similar to the reimbursement of TARE in CRLM, more weight is given to the MDB to decide on reimbursable treatment pathways.

Considering the 2022 BCLC guidelines [3] that recommend that BCLC 0 or BCLC A patients could receive TARE if neither ablation, resection, nor TACE is feasible [49], Group 2a was selected to assess the benefit of TARE Y90 in this subpopulation compared to more advanced-stage patients in Group 2b. Our analysis showed that Group 2a had better survival outcomes than patients included in Group 2b (*p* = 0.026, Fig. 3B) which, along with evidence from other large prospective studies where median OS for BCLC A subgroups treated with TARE was not reached during the course of the study [13, 58], confirm the clinical value of using TARE in early-stage HCC patients.

In a broader context, differences in reimbursement criteria for TARE across European countries add a layer of complexity to the landscape of liver cancer treatment. French reimbursement of eligible medical devices requires an exhaustive evaluation of safety and effectiveness of the technologies, which was the case for Y90 resin microspheres. To our knowledge, this is the only system requiring periodic re-evaluations of the available clinical evidence to maintain reimbursement. In the UK, the National Institute for Health and Care Excellence (NICE) also considers economic aspects by assessing cost-effectiveness profile and budget impact, and recommended TARE using SIR-Spheres in HCC patients with Child–Pugh A for whom conventional transarterial therapies were deemed inappropriate [59], which still limits their use to at least intermediate stages. German recommendations include the treatment with Y90 resin microspheres of early-stage patients based on their use as bridging or downstaging therapy in patients within Milan Criteria and with preserved liver function [60], which aligns with our findings on BCLC A patients showing better survival compared to more advanced patients.

Considering the importance of the HAS criteria in reimbursement eligibility and the fact that one key area of their assessment was effectiveness, a multivariable analysis on the whole HCC and CRLM groups was performed to assess the HAS criteria in our cohort and identify new potential factors that are strongly related to the effectiveness of TARE.

In the HCC cohort, the HAS criteria included patients with preserved liver function (Child–Pugh A or B), which is consistent with our results of Child–Pugh B or C (HR 1.8, 95% CI 1.2–2.9) being associated with shorter survival (Table 2). Our multivariable analysis also found hepatic tumour burden > 25% as an independent indicator of shorter survival (HR 1.7, 95% CI 1.1–1.7) (Table 2), PFS (*p* = 0.001) and hPFS (*p* = 0.021) (Supplementary Table 2), pointing out the importance of considering tumour burden in patient selection. Additionally, dose methodology using partition model (HR 0.6, 95% CI 0.4–0.9) and post-TARE downstaging (HR 0.2, 95% 0.1–0.5) appeared to be strong predictors for longer OS (Table 2). These results were previously reported [48, 61] and underline the value of personalised treatments and the impact of liver status on treatment effectiveness.

As expected, distinct prognostic factors were observed in the CRLM cohort. Extrahepatic disease (HR 4.0, 95% 1.2–13.5), more than 5 nodules (HR 2.8, 95% 0.8–9.5), presence of bilobar disease (HR 4.0, 95% 1.3–12.5) and ALBI score 3 (HR 147.7, 95% 8.5–2566.2) were factors associated with shorter survival (Table 2). Other studies have also reported the association between short survival and tumour burden in CRLM patients [18], but our cohort did not confirm this. However, it is directly related to our findings on the number of nodules and bilobar location. Regarding liver function, ALBI is a well-documented prognostic parameter in HCC cohorts, which has been proven helpful in predicting outcomes in patients treated with TARE [62–64]. In our study, ALBI score 3 was strongly associated with shorter survival (*p* = 0.001) in the CRLM group, which suggests that measuring these laboratory values could also be beneficial for optimal patient selection in this sub-cohort.

In this prospective study, safety results display low toxicity and excellent safety profile, which has been acknowledged by previous studies [13, 15, 19, 65–67]. Influence on HRQoL was minimal, which reinforces findings from previous randomised controlled trials on TARE using Y90 resin microspheres showing that TARE was better tolerated than sorafenib [57] and achieved similar quality-of-life results as atezolizumab–bevacizumab [68]. Indeed, TARE is characterised by its ability to maintain a stable quality-of-life outcome [61, 69, 70] and is considered favourable to TACE in that respect [71–73].

## Limitations

Limitations of this study include those inherent to real-world, non-interventional study designs, such as a heterogeneous patient population, centre bias and other potential bias due to confounding factors that were not accounted for. Real-world data studies are powerful tools to evaluate real-life clinical applications and understand how treatments are applied in practice. However, this comes with limitations that we acknowledge, specifically for tumour control evaluation. There was significant heterogeneity in the methods used, Positron Emission Tomography Response Criteria In Solid Tumours (PERCIST), Response Evaluation Criteria In Solid Tumours (RECIST) and (modified) RECIST, across and within sites, which, unfortunately, withstood any option to assess tumour control.

## Conclusion

Our prospective, multicentre, observational study on primary and secondary liver tumours confirms that TARE using Y90 resin microspheres is an effective treatment that can be safely applied to patients as decided by multidisciplinary tumour boards (MDBs). No significant differences in safety or effectiveness were found between Group 1 vs Group 2a and Group 3 vs Group 4a, suggesting that instead of restrictive reimbursement criteria, more decision-making power could be given to MDB.

## Supplementary Information

Below is the link to the electronic supplementary material.

Supplementary file1 (DOCX 87 KB)

## Footnotes

1 Eastern Cooperative Oncology Group.

2 Barcelona Clinic Liver Cancer.
