# Supplementary material for: Transarterial Radioembolisation with Y90 Resin Microspheres and the Effect of Reimbursement Criteria in France: Final Results of the CIRT-FR Prospective Observational Study
Source: Cardiovasc Intervent Radiol. 2025 Jan 14;48(2):205–20. doi: 10.1007/s00270-024-03955-y (PMC11790776; doi:10.1007/s00270-024-03955-y)
Supplement: Supplementary file 1 — Supplementary file1 (DOCX 87 KB) [file 270_2024_3955_MOESM1_ESM.docx]

**Supplements to:**

Transarterial radioembolization with Y90 resin microspheres in France: final results of the CIRT-FR prospective observational study

Contents

[Supplementary Table 1: Treatment characteristics 2](#_Toc176185913)

[Supplementary Table 2: Effectiveness (PFS, hPFS) by main indication 4](#_Toc176185914)

[Supplementary Table 3: Adverse event type summary 6](#_Toc176185915)

[Supplementary Table 4A: Baseline characteristics comparison HCC 7](#_Toc176185916)

[Supplementary Table 4B: Baseline characteristics comparison CRLM 10](#_Toc176185917)

[Supplementary Table 5A: Functional scale score by analysis group: HCC cohort 12](#_Toc176185918)

[Supplementary Table 5B: Symptom scale score by analysis group: HCC cohort 14](#_Toc176185919)

[Supplementary Table 5C: Global health score by analysis group: HCC cohort 18](#_Toc176185920)

# Supplementary Table 1: Treatment characteristics

|  | **HCC** | **CRLM** |
| --- | --- | --- |
| **Total N (%)** | | |
|  | 193 (58.8) | 59 (18.0) |
| **Number of treatment sessions** | | |
| 1 | 150 (77.7) | 54 (91.5) |
| 2 | 36 (18.7) | 4 (6.8) |
| 3 | 7 (3.6) | 1 (1.7) |
| **BSA (m2)** | | |
| Median (IQR) | 1.9 (1.8 - 2.1) | 1.8 (1.6 - 1.9) |
| **Prescribed activity (GBq)** | | |
| Median (IQR) | 1.0 (0.7 - 1.6) | 1.5 (1.0 - 1.7) |
| **Delivered activity (GBq)** | | |
| Median (IQR) | 1.0 (0.7 - 1.6) | 1.5 (1.0 - 1.7) |
| **Liver treatment target** | | |
| Left lobe | 50 (25.9) | 4 (6.8) |
| Right lobe | 105 (54.4) | 20 (33.9) |
| Sequential | 20 (10.4) | 5 (8.5) |
| Whole liver (sequential) | 10 (5.2) | 0 (0) |
| Whole liver (single catheter) | 2 (1.0) | 9 (15.3) |
| Whole liver (split administration) | 6 (3.1) | 21 (35.6) |
| **Prescribed to whole liver or separate halves** | | |
| Right and left | 59 (30.6) | 37 (62.7) |
| Unknown | 10 (5.2) | 0 (0) |
| Whole | 124 (64.2) | 22 (37.3) |
| **Delivered activity within 90% of prescribed activity** | | |
| No | 2 (1.0) | 1 (1.7) |
| Yes | 191 (99.0) | 58 (98.3) |
| **Hospital stay (1st treatment)** | | |
| < 24 hours | 3 (1.6) | 0 (0) |
| 24 to <36 hours | 35 (18.1) | 13 (22.0) |
| 36 to <48 hours | 32 (16.6) | 23 (39.0) |
| 48 to <72 hours | 97 (50.3) | 11 (18.6) |
| 72+ hours | 26 (13.5) | 11 (18.6) |
| Unknown | 0 (0) | 1 (1.7) |
| **Bilirubin (µmol/L)** | | |
| Median (IQR) | 13.0 (8.5 - 21.0) | 9.0 (6.7 - 13.0) |
| **Creatinine (µmol/L)** | | |
| Median (IQR) | 74.0 (63.0 - 93.8) | 67.5 (56.0 - 75.0) |
| **Albumin (g/L)** | | |
| Median (IQR) | 34.0 (30.0 - 37.0) | 36.0 (31.1 - 39.0) |
| **ALT (U/L)** | | |
| Median (IQR) | 35.0 (24.0 - 50.0) | 32.0 (23.0 - 56.0) |
| **AST (U/L)** | | |
| Median (IQR) | 49.0 (35.0 - 73.0) | 39.0 (26.8 - 73.5) |
| **Platelets (109/L)** | | |
| Median (IQR) | 157.0 (102.2 - 231.5) | 195.0 (172.0 - 244.0) |
| **INR** | | |
| Median (IQR) | 1.1 (1.0 - 1.2) | 1.0 (1.0 - 1.1) |
| **Post TARE procedures** | | |
| No post TARE procedures | 126 (65.3) | 49 (83.1) |
| Surgical procedures | 12 (20.0) | 2 (40.0) |
| Ablative procedures | 11 (18.3) | 1 (20.0) |
| Vascular procedures | 33 (55.0) | 3 (60.0) |
| Radiotherapy | 15 (25.0) | 0 (0) |
| **Post systemic chemotherapy** | | |
| No | 119 (61.7) | 10 (16.9) |
| Unknown | 7 (3.6) | 5 (8.5) |
| Yes | 67 (34.7) | 44 (74.6) |
| **Total volume (cc) - overall** | | |
| Median (IQR) | 1782.5 (1411.0 - 2142.0) | 1595.7 (1368.5 - 1875.0) |
| **Tumor volume (cc) - overall** | | |
| Median (IQR) | 142.0 (53.0 - 411.5) | 149.0 (71.0 - 419.0) |
| **All liver tumors targeted** | | |
| No | 61 (31.6) | 14 (23.7) |
| Yes | 131 (67.9) | 44 (74.6) |
| Unknown | 1 (0.5) | 1 (1.7) |
| *Abbreviations. HCC: hepatocellular carcinoma; CRLM: colorectal liver metastases; GBq: giga-becquerel; IQR: interquartile range; ALT: alanine aminotransferase; AST: aspartate aminotransferase; INR: international normalized ratio; cc: cubic centimeter.* | | |

# Supplementary Table 2: Effectiveness (PFS, hPFS) by main indication

|  |  | | |  | **Median PFS/hPFS (months, 95% CI)** | | | **Events** | | **Censored** | | **HR (95% CI)** | | **P value** |
| --- | --- | --- | --- | --- | --- | --- | --- | --- | --- | --- | --- | --- | --- | --- |
| **HCC – Progression-free survival** | | | | | | | | | | | | | | |
|  | Overall | | | | | | | | | | | | | |
|  |  | | | Whole cohort | | 7.4 (6.3-9.0) | | 174 (90.2) | | 19 (9.8) | | - | | - |
|  | Tumor burden | | | | | | | | | | | | | |
|  |  | | | ≤ 25% | | 8.2 (6.6-9.7) | | 130 (89.0) | | 16 (11.0) | | - | | - |
|  |  | | | >25% | | 6.0 (3.5-7.8) | | 36 (97.3) | | 1 (2.7) | | 2.0 (1.3-2.9) | | 0.001 |
|  | Prior surgery | | | | | | | | | | | | | |
|  |  | | | No | | 8.0 (6.5-9.3) | | 154 (89.0) | | 19 (11.0) | | - | | - |
|  |  | | | Yes | | 6.2 (2.8-8.1) | | 20 (100.0) | | 0 (0) | | 1.9 (1.1-3.1) | | 0.018 |
|  | Prior adjuvant chemotherapy | | | | | | | | | | | | | |
|  |  | | | No | | 7.6 (6.4-9.0) | | 172 (90.1) | | 19 (9.9) | | - | | - |
|  |  | | | Yes | | 2.9 (2.5-3.2) | | 2 (100.0) | | 0 (0) | | 19.4 (2.5-151.2) | | 0.005 |
|  | Post TARE downstaging | | | | | | | | | | | | | |
|  |  | | | No | | 6.7 (6.1-8.6) | | 153 (92.7) | | 12 (7.3) | | - | | - |
|  |  | | | Yes | | 16.9 (8.8-28.1) | | 15 (71.4) | | 6 (28.6) | | 0.5 (0.3-0.8) | | 0.006 |
| **HCC – Hepatic progression-free survival** | | | | | | | | | | | | | | |
|  | Overall | | | | | | | | | | | | | |
|  |  | | | Whole cohort | | 8.1 (6.5-9.4) | | 172 (89.1) | | 21 (10.9) | | - | | - |
|  | Tumor burden | | | | | | | | | | | | | |
|  |  | | | ≤ 25% | | 8.6 (6.8-10.9) | | 128 (87.7) | | 18 (12.3) | | - | | - |
|  |  | | | >25% | | 6.0 (3.8-8.9) | | 36 (97.3) | | 1 (2.7) | | 1.7 (1.1-2.6) | | 0.021 |
|  | Prior adjuvant chemotherapy | | | | | | | | | | | | | |
|  |  | | | No | | 8.2 (6.7-9.7) | | 170 (89.0) | | 21 (11.0) | | - | | - |
|  |  | | | Yes | | 2.9 (2.5-3.2) | | 2 (100.0) | | 0 (0) | | 21.6 (2.6-175.9) | | 0.004 |
|  | Post TARE downstaging | | | | | | | | | | | | | |
|  |  | | | No | | 7.2 (6.3-9.0) | | 153 (92.7) | | 12 (7.3) | | - | | - |
|  |  | | | Yes | | 20.5 (10.5-NA) | | 13 (61.9) | | 8 (38.1) | | 0.4 (0.2-0.8) | | 0.005 |
| **CRLM – Progression-free Survival** | | | | | | | | | | | | | | |
|  | Overall | | | | | | | | | | | | | |
|  |  | | | Whole cohort | | 3.2 (2.3-4.0) | | 55 (93.2) | | 4 (6.8) | | - | | - |
|  | Extrahepatic disease | | | | | | | | | | | | | |
|  |  | | | No | | 3.9 (3.1-5.1) | | 34 (91.9) | | 3 (8.1) | | - | | - |
|  |  | | | Yes | | 2.0 (1.2-3.4) | | 21 (95.5) | | 1 (4.5) | | 3.7 (1.9-7.3) | | <0.001 |
|  | Tumor burden | | | | | | | | | | | | | |
|  |  | | | ≤ 25% | | 3.4 (2.6-5.0) | | 35 (89.7) | | 4 (10.3) | | - | | - |
|  |  | | | >25% | | 2.1 (0.9-.35) | | 15 (100.0) | | 0 (0) | | 3.1 (1.6-6.1) | | 0.001 |
| **CRLM – Hepatic progression-free survival** | | | | | | | | | | | | | | |
|  | Overall | | | | | | | | | | | | | |
|  |  | | | Whole cohort | | 4.0 (3.1-5.5) | | 55 (93.2) | | 4 (6.8) | | - | | - |
|  | Tumor burden | | | | | | | | | | | | | |
|  |  | | | ≤ 25% | | 5.0 (3.1-6.4) | | 35 (89.7) | | 4 (10.3) | | - | | - |
|  |  | | | >25% | | 3.1 (0.9-4.0) | | 15 (100.0) | | 0 (0) | | 2.4 (1.0-5.9) | | 0.006 |
|  | | ALBI Score | | | | | | | | | | | | |
|  | |  | 1 | | | | 5.2 (2.1-10.8) | | 15 (100.0) | | 0 (0) | | - | - |
|  | |  | 3 | | | | 0.9 (0.7-3.5) | | 4 (100.0) | | 0 (0) | | 4.7 (1.3-17.5) | 0.022 |
| *Abbreviations. HCC: hepatocellular carcinoma; CRLM: colorectal liver metastases; CI: confidence interval; (h)PFS: (hepatic) progression-free survival; HR: hazard ratio; ALBI: albumin-bilirubin.* | | | | | | | | | | | | | | |

# Supplementary Table 3: Adverse event type summary

|  |  | **Unknown** | **Grade 1** | **Grade 2** | **Grade 3** | **Grade 4** |
| --- | --- | --- | --- | --- | --- | --- |
| **HCC** | | | | | | |
| Patients with any AEs | | 22 (11.4) | 15 (7.8) | 18 (9.3) | 16 (8.3) | 5 (2.6) |
| Total number of AEs | | 90 | 67 | 41 | 25 | 6 |
|  | Abdominal Pain | 12 (6.2) | 3 (1.6) | 2 (1) | 2 (1) |  |
|  | Analytical | 3 (1.6) | 1 (0.5) | 2 (1) |  |  |
|  | Bleeding | 3 (1.6) | 1 (0.5) |  |  | 1 (0.5) |
|  | Cardio-pulmonary | 5 (2.6) |  | 1 (0.5) |  | 1 (0.5) |
|  | Cutaneous complications and alopecia | 2 (1) | 3 (1.6) | 1 (0.5) |  |  |
|  | Digestive | 3 (1.6) | 6 (3.1) | 4 (2.1) | 4 (2.1) |  |
|  | Fatigue | 9 (4.7) | 13 (6.7) | 10 (5.2) | 5 (2.6) | 1 (0.5) |
|  | Fever | 3 (1.6) | 1 (0.5) |  | 2 (1) |  |
|  | GI Ulceration |  |  | 1 (0.5) |  |  |
|  | Gastritis |  |  | 2 (1) |  |  |
|  | General | 8 (4.1) | 6 (3.1) | 3 (1.6) | 3 (1.6) | 1 (0.5) |
|  | Infectious | 1 (0.5) |  |  |  |  |
|  | Liver and portal system | 10 (5.2) | 1 (0.5) | 4 (2.1) | 5 (2.6) | 2 (1) |
|  | Nausea | 2 (1) | 1 (0.5) | 1 (0.5) |  |  |
|  | Neurological, pain, and other sensitive disorders | 5 (2.6) | 6 (3.1) | 2 (1) | 3 (1.6) |  |
|  | Radiation Pneumonitis |  | 1 (0.5) |  |  |  |
|  | Renal and fluid balance | 2 (1) |  |  |  |  |
|  | Vomiting | 3 (1.6) |  |  |  |  |
| **CRLM** | | | | | | |
| Patients with any AEs | | 1 (1.7) | 1 (1.7) | 3 (5.1) | 1 (1.7) |  |
| Total number of AEs | | 3 | 4 | 3 | 2 |  |
|  | Abdominal Pain |  | 1 (1.7) |  |  |  |
|  | Digestive |  | 1 (1.7) |  |  |  |
|  | Fatigue |  | 1 (1.7) | 1 (1.7) |  |  |
|  | Fever |  |  | 1 (1.7) |  |  |
|  | General | 2 (3.4) | 1 (1.7) |  |  |  |
|  | Liver and portal system | 1 (1.7) |  | 1 (1.7) | 1 (1.7) |  |
| *Abbreviations. HCC: hepatocellular carcinoma; CRLM: colorectal liver metastases; AE: adverse event; GI: gastrointestinal* | | | | | | |

# Supplementary Table 4A: Baseline characteristics comparison HCC

|  | **Group1** | **Group2** | **P value** |
| --- | --- | --- | --- |
| **Total N (%)** |  |  |  |
|  | 144 (75.8) | 46 (24.2) |  |
| **Sex** |  |  |  |
| Female | 16 (11.1) | 9 (19.6) | 0.220 |
| Male | 128 (88.9) | 37 (80.4) |  |
| **ECOG performance status** |  |  |  |
| 0 | 61 (42.4) | 24 (52.2) | <0.001 |
| 1 | 83 (57.6) | 9 (19.6) |  |
| 2 | 0 (0.0) | 11 (23.9) |  |
| 3 | 0 (0.0) | 1 (2.2) |  |
| (Missing) | 0 (0.0) | 1 (2.2) |  |
| **BCLC mDerived** |  |  |  |
| A | 0 (0.0) | 21 (45.7) | <0.001 |
| B | 27 (18.8) | 0 (0.0) |  |
| C | 117 (81.2) | 17 (37.0) |  |
| D | 0 (0.0) | 8 (17.4) |  |
| **Child-Pugh Class Derived** |  |  |  |
| A (5-6) | 102 (70.8) | 28 (60.9) | <0.001 |
| B (7-8) | 23 (16.0) | 8 (17.4) |  |
| C (9+) | 0 (0.0) | 7 (15.2) |  |
| Unknown | 19 (13.2) | 3 (6.5) |  |
| **Cirrhosis** |  |  |  |
| No | 34 (23.6) | 14 (30.4) | 0.464 |
| Yes | 110 (76.4) | 32 (69.6) |  |
| **Ascites** |  |  |  |
| No | 140 (97.2) | 44 (95.7) | 0.963 |
| Yes | 4 (2.8) | 2 (4.3) |  |
| **Extrahepatic disease** |  |  |  |
| No | 117 (81.2) | 41 (89.1) | 0.309 |
| Yes | 27 (18.8) | 5 (10.9) |  |
| **Portal vein thrombosis status** |  |  |  |
| Lobar Thrombosis | 16 (11.1) | 1 (2.2) | <0.001 |
| Main Thrombosis | 0 (0.0) | 7 (15.2) |  |
| Patent | 82 (56.9) | 32 (69.6) |  |
| Segmental Thrombosis | 46 (31.9) | 6 (13.0) |  |
| **Prior systemic chemotherapy lines** |  |  |  |
| 1 | 13 (9.0) | 0 (0.0) | 0.220 |
| 2 | 2 (1.4) | 2 (4.3) |  |
| 3 | 1 (0.7) | 0 (0.0) |  |
| No prior systemic therapy | 128 (88.9) | 44 (95.7) |  |
| **Liver tumor location** |  |  |  |
| Bilobar | 41 (28.5) | 7 (15.2) | 0.179 |
| Left | 28 (19.4) | 12 (26.1) |  |
| Right | 75 (52.1) | 27 (58.7) |  |
| **Number of tumors** |  |  |  |
| 1 | 58 (40.3) | 31 (67.4) | 0.091 |
| 2 | 23 (16.0) | 4 (8.7) |  |
| 3 | 21 (14.6) | 5 (10.9) |  |
| 4 | 12 (8.3) | 1 (2.2) |  |
| 5 | 5 (3.5) | 0 (0.0) |  |
| 6 | 4 (2.8) | 0 (0.0) |  |
| 7 | 1 (0.7) | 0 (0.0) |  |
| 8 | 1 (0.7) | 0 (0.0) |  |
| 9 | 1 (0.7) | 0 (0.0) |  |
| 10 | 0 (0.0) | 0 (0.0) |  |
| 10+ | 5 (3.5) | 0 (0.0) |  |
| Uncountable | 13 (9.0) | 4 (8.7) |  |
| Unknown | 0 (0.0) | 1 (2.2) |  |
| **Hepatic tumor burden** |  |  |  |
| ≤ 25% | 111 (77.1) | 33 (71.7) | 0.518 |
| >25% | 26 (18.1) | 11 (23.9) |  |
| (Missing) | 7 (4.9) | 2 (4.3) |  |
| **Lung shunt (%)** |  |  |  |
| >10% to 15% | 1 (0.7) | 0 (0.0) | 0.740 |
| >15% to 20% | 3 (2.1) | 0 (0.0) |  |
| >20% | 1 (0.7) | 0 (0.0) |  |
| 0 to 10% | 138 (95.8) | 46 (100.0) |  |
| Unknown | 1 (0.7) | 0 (0.0) |  |
| **Method for determining dose** |  |  |  |
| BSA | 17 (11.8) | 7 (15.2) | 0.291 |
| Empiric | 0 (0.0) | 0 (0.0) |  |
| Modified BSA | 25 (17.4) | 3 (6.5) |  |
| Other | 1 (0.7) | 0 (0.0) |  |
| Partition | 101 (70.1) | 36 (78.3) |  |
| **Concomitant chemotherapy** |  |  |  |
| No | 136 (94.4) | 46 (100.0) | 0.226 |
| Yes | 8 (5.6) | 0 (0.0) |  |
| **ALBI grade** |  |  |  |
| 1 | 18 (12.5) | 4 (8.7) | 0.203 |
| 2 | 101 (70.1) | 25 (54.3) |  |
| 3 | 5 (3.5) | 4 (8.7) |  |
| (Missing) | 20 (13.9) | 13 (28.3) |  |
| **Intent of SIR-Spheres therapy** |  |  |  |
| Ablation (Radiation Segmentectomy) | 13 (9.0) | 2 (4.3) | 0.675 |
| Bridge to Liver Transplant | 15 (10.4) | 4 (8.7) |  |
| Down-Sizing/Down-Staging | 12 (8.3) | 3 (6.5) |  |
| Palliative (Cytoreduction) | 104 (72.2) | 37 (80.4) |  |
| **Chemo-refractory or intolerant** |  |  |  |
| No | 136 (94.4) | 44 (95.7) | 1.000 |
| Yes | 8 (5.6) | 2 (4.3) |  |
| *Abbreviations. HCC: hepatocellular carcinoma; IQR: interquartile range; ECOG: Eastern Cooperative Oncology Group; BCLC: Barcelona Clinic Liver Cancer; (m)BSA: (modified) body surface area; ALBI: Albumin-Bilirubin.* | | | |

# Supplementary Table 4B: Baseline characteristics comparison CRLM

|  | **Group 3** | **Group 4** | **P value** |
| --- | --- | --- | --- |
| **Total N (%)** |  |  |  |
|  | 10 (17.5) | 47 (82.5) |  |
| **Sex** |  |  |  |
| Female | 2 (20.0) | 23 (48.9) | 0.186 |
| Male | 8 (80.0) | 24 (51.1) |  |
| **ECOG performance status** |  |  |  |
| 0 | 3 (30.0) | 19 (45.2) | 0.769 |
| 1 | 6 (60.0) | 19 (45.2) |  |
| 2 | 1 (10.0) | 3 (7.1) |  |
| 3 |  | 1 (2.4) |  |
| **Extrahepatic disease** |  |  |  |
| No | 10 (100.0) | 25 (53.2) | 0.016 |
| Yes |  | 22 (46.8) |  |
| **Portal vein thrombosis status** |  |  |  |
| Lobar Thrombosis | 1 (10.0) |  | 0.148 |
| Main Thrombosis | 0 (0.0) | 3 (6.4) |  |
| Patent | 8 (80.0) | 39 (83.0) |  |
| Segmental Thrombosis | 1 (10.0) | 5 (10.6) |  |
| **Prior systemic chemotherapy lines** |  |  |  |
| 1 | 3 (30.0) | 4 (8.5) | 0.238 |
| 2 | 5 (50.0) | 22 (46.8) |  |
| 3 | 2 (20.0) | 19 (22.2) |  |
| No prior systemic therapy | 0 (0.0) | 2 (4.3) |  |
| **Liver tumor location** |  |  |  |
| Bilobar | 6 (60.0) | 30 (63.8) | 0.972 |
| Left | 1 (10.0) | 4 (8.5) |  |
| Right | 3 (30.0) | 13 (27.7) |  |
| **Number of tumors** |  |  |  |
| 1 | 0 (0.0) | 7 (14.9) | 0.148 |
| 2 | 2 (20.0) | 1 (2.1) |  |
| 3 | 2 (20.0) | 5 (10.6) |  |
| 4 | 1 (10.0) | 3 (6.4) |  |
| 5 | 0 (0.0) | 4 (8.5) |  |
| 6 | 4 (2.8) | 0 (0.0) |  |
| 7 | 1 (10.0) | 0 (0.0) |  |
| 8 | 0 (0.0) | 0 (0.0) |  |
| 9 | 0 (0.0) | 1 (2.1) |  |
| 10 | 0 (0.0) | 2 (4.3) |  |
| 10+ | 2 (20.0) | 1 (2.1) |  |
| Uncountable | 4 (40.0) | 14 (29.8) |  |
| Unknown | 0 (0.0) | 2 (4.3) |  |
| **Hepatic tumor burden** |  |  |  |
| ≤ 25% | 10 (100.0) | 27 (64.3) | 0.064 |
| >25% | 0 (0.0) | 15 (35.7) |  |
| **Method for determining dose** |  |  |  |
| BSA | 6 (60.0) | 19 (40.4) | 0.812 |
| Empiric |  | 1 (2.1) |  |
| Modified BSA | 1 (10.0) | 3 (6.4) |  |
| Other |  | 3 (6.4) |  |
| Partition | 3 (30.0) | 20 (42.6) |  |
| Unknown |  | 1 (2.1) |  |
| **Concomitant chemotherapy** |  |  |  |
| No | 8 (80.0) | 37 (78.7) | 1.000 |
| Yes | 2 (20.0) | 10 (21.3) |  |
| **ALBI grade** |  |  |  |
| 1 | 2 (25.0) | 12 (35.3) | 0.428 |
| 2 | 6 (75.0) | 18 (52.9) |  |
| 3 | 0 (0.0) | 4 (11.8) |  |
| **Intent of SIR-Spheres therapy** |  |  |  |
| Ablation (Radiation Segmentectomy) | 0 (0.0) | 3 (6.4) | 0.192 |
| Bridge to Liver Transplant | 0 (0.0) | 2 (4.3) |  |
| Down-Sizing/Down-Staging | 0 (0.0) | 11 (23.4) |  |
| Palliative (Cytoreduction) | 10 (100.0) | 31 (66.0) |  |
| **Chemo-refractory or intolerant** |  |  |  |
| No | 0 (0.0) | 30 (63.8) | 0.001 |
| Yes | 10 (100.0) | 17 (36.2) |  |
| *Abbreviations. CRLM: colorectal liver metastases; IQR: interquartile range; ECOG: Eastern Cooperative Oncology Group; (m)BSA: (modified) body surface area; ALBI: Albumin-Bilirubin.* | | | |

# Supplementary Table 5A: Functional scale score by analysis group: HCC cohort

|  | | **Improved** | **Stable** | **Deteriorated** | **P Value** |
| --- | --- | --- | --- | --- | --- |
| Overall functional scale | | | | | |
| Month 3 | Group1  n=58 | 5 (8.6) | 42 (72.4) | 11 (19) | 0.399 |
|  | Group2  n=26 | 3 (11.5) | 15 (57.7) | 8 (30.8) |  |
| Month 6 | Group1  n=31 | 6 (19.4) | 21 (67.7) | 4 (12.9) | 0.080 |
|  | Group2  n=15 | 0 (0) | 10 (66.7) | 5 (33.3) |  |
| Month 9 | Group1  n=25 | 2 (8) | 14 (56) | 9 (36) | 0.780 |
|  | Group2  n=14 | 2 (14.3) | 8 (57.1) | 4 (28.6) |  |
| Month 12 | Group1  n=23 | 0 (0) | 15 (65.2) | 8 (34.8) |  |
|  | Group2  n=7 | 0 (0) | 4 (57.1) | 3 (42.9) |  |
| Physical | | | | | |
| Month 3 | Group1  n=63 | 8 (12.7) | 44 (69.8) | 11 (17.5) | 0.016 |
|  | Group2  n=26 | 0 (0) | 15 (57.7) | 11 (42.3) |  |
| Month 6 | Group1  n=33 | 7 (21.2) | 18 (54.5) | 8 (24.2) | 0.213 |
|  | Group2  n=15 | 1 (6.7) | 7 (46.7) | 7 (46.7) |  |
| Month 9 | Group1  n=26 | 2 (7.7) | 15 (57.7) | 9 (34.6) | 0.996 |
|  | Group2  n=14 | 1 (7.1) | 8 (57.1) | 5 (35.7) |  |
| Month 12 | Group1  n=22 | 2 (9.1) | 13 (59.1) | 7 (31.8) | 0.409 |
|  | Group2  n=7 | 0 (0) | 3 (42.9) | 4 (57.1) |  |
| Cognitive | | | | | |
| Month 3 | Group1  n=58 | 10 (17.2) | 33 (56.9) | 15 (25.9) | 0.210 |
|  | Group2  n=25 | 2 (8) | 12 (48) | 11 (44) |  |
| Month 6 | Group1  n=31 | 6 (19.4) | 19 (61.3) | 6 (19.4) | 0.564 |
|  | Group2  n=15 | 2 (13.3) | 8 (53.3) | 5 (33.3) |  |
| Month 9 | Group1  n=25 | 2 (8) | 8 (32) | 15 (60) | 0.458 |
|  | Group2  n=13 | 0 (0) | 6 (46.2) | 7 (53.8) |  |
| Month 12 | Group1  n=23 | 3 (13) | 10 (43.5) | 10 (43.5) | 0.128 |
|  | Group2  n=6 | 1 (16.7) | 0 (0) | 5 (83.3) |  |
| Role | | | | | |
| Month 3 | Group1  n=63 | 7 (11.1) | 34 (54) | 22 (34.9) | 0.317 |
|  | Group2  n=26 | 6 (23.1) | 11 (42.3) | 9 (34.6) |  |
| Month 6 | Group1  n=33 | 5 (15.2) | 21 (63.6) | 7 (21.2) | 0.093 |
|  | Group2  n=15 | 0 (0) | 8 (53.3) | 7 (46.7) |  |
| Month 9 | Group1  n=26 | 2 (7.7) | 13 (50) | 11 (42.3) | 0.781 |
|  | Group2  n=14 | 2 (14.3) | 7 (50) | 5 (35.7) |  |
| Month 12 | Group1  n=21 | 2 (9.5) | 12 (57.1) | 7 (33.3) | 0.444 |
|  | Group2  n=7 | 0 (0) | 3 (42.9) | 4 (57.1) |  |
| Social | | | | | |
| Month 3 | Group1  n=58 | 8 (13.8) | 34 (58.6) | 16 (27.6) | 0.124 |
|  | Group2  n=26 | 8 (30.8) | 10 (38.5) | 8 (30.8) |  |
| Month 6 | Group1  n=31 | 6 (19.4) | 18 (58.1) | 7 (22.6) | 0.285 |
|  | Group2  n=15 | 5 (33.3) | 5 (33.3) | 5 (33.3) |  |
| Month 9 | Group1  n=25 | 3 (12) | 15 (60) | 7 (28) | 0.554 |
|  | Group2  n=15 | 3 (21.4) | 6 (42.9) | 5 (35.7) |  |
| Month 12 | Group1  n=23 | 2 (8.7) | 12 (52.2) | 9 (39.1) | 0.007 |
|  | Group2  n=7 | 4 (57.1) | 0 (0) | 3 (42.9) |  |
| *Abbreviations. HAS: Haute Autorité de Santé; HCC: hepatocellular carcinoma.* | | | | | |

# Supplementary Table 5B: Symptom scale score by analysis group: HCC cohort

|  | | **Improved** | **Stable** | **Deteriorated** | **P value** |
| --- | --- | --- | --- | --- | --- |
| Symptom scale score | | | | | |
| Month 3 | Group 1  n=63 | 6 (9.5) | 37 (58.7) | 20 (31.7) | 0.069 |
|  | Group 2  n=26 | 1 (3.8) | 10 (38.5) | 15 (57.7) |  |
| Month 6 | Group 1  n=33 | 6 (18.2) | 19 (57.6) | 8 (24.2) | 0.532 |
|  | Group 2  n=15 | 1 (6.7) | 9 (60) | 5 (33.3) |  |
| Month 9 | Group 1  n=26 | 5 (19.2) | 15 (57.7) | 6 (23.1) | 0.803 |
|  | Group 2  n=14 | 3 (21.4) | 9 (64.3) | 2 (14.3) |  |
| Month 12 | Group 1  n=22 | 5 (22.7) | 11 (50) | 6 (27.3) | 0.221 |
|  | Group 2  n=7 | 0 (0) | 3 (42.9) | 4 (57.1) |  |
| Fatigue symptom scale score | | | | | |
| Month 3 | Group 1  n=63 | 15 (23.8) | 16 (25.4) | 32 (50.8) | 0.544 |
|  | Group 2  n=26 | 6 (23.1) | 4 (15.4) | 16 (61.5) |  |
| Month 6 | Group 1  n=33 | 9 (27.3) | 13 (39.4) | 11 (33.3) | 0.054 |
|  | Group 2  n=14 | 2 (14.3) | 2 (14.3) | 10 (71.4) |  |
| Month 9 | Group 1  n=26 | 7 (26.9) | 9 (34.6) | 10 (38.5) | 0.466 |
|  | Group 2  n=14 | 6 (42.9) | 5 (35.7) | 3 (21.4) |  |
| Month 12 | Group 1  n=22 | 7 (31.8) | 7 (31.8) | 8 (36.4) | 0.157 |
|  | Group 2  n=7 | 2 (28.6) | 0 (0) | 5 (71.4) |  |
| Nausea/Vomiting symptom scale score | | | | | |
| Month 3 | Group 1  n=63 | 2 (3.2) | 44 (69.8) | 17 (27) | 0.987 |
|  | Group 2  n=26 | 1 (3.8) | 18 (69.2) | 7 (26.9) |  |
| Month 6 | Group 1  n=32 | 1 (3.1) | 27 (84.4) | 4 (12.5) | 0.384 |
|  | Group 2  n=15 | 1 (6.7) | 10 (66.7) | 4 (26.7) |  |
| Month 9 | Group 1  n=26 | 1 (3.8) | 22 (84.6) | 3 (11.5) | 0.557 |
|  | Group 2  n=14 | 0 (0) | 11 (78.6) | 3 (21.4) |  |
| Month 12 | Group 1  n=22 | 1 (4.5) | 17 (77.3) | 4 (18.2) | 0.734 |
|  | Group 2  n=7 | 0 (0) | 5 (71.4) | 2 (28.6) |  |
| Pain symptom scale score | | | | | |
| Month 3 | Group 1  n=63 | 14 (22.2) | 29 (46) | 20 (31.7) | 0.027 |
|  | Group 2  n=26 | 2 (7.7) | 8 (30.8) | 16 (61.5) |  |
| Month 6 | Group 1  n=33 | 6 (18.2) | 18 (54.5) | 9 (27.3) | 0.479 |
|  | Group 2  n=15 | 5 (33.3) | 6 (40) | 4 (26.7) |  |
| Month 9 | Group 1  n=26 | 5 (19.2) | 14 (53.8) | 7 (26.9) | 0.972 |
|  | Group 2  n=14 | 3 (21.4) | 7 (50) | 4 (28.6) |  |
| Month 12 | Group 1  n=23 | 4 (17.4) | 12 (52.2) | 7 (30.4) | 0.542 |
|  | Group 2  n=7 | 2 (28.6) | 2 (28.6) | 3 (42.9) |  |
| Dyspnea scale score | | | | | |
| Month 3 | Group 1  n=61 | 7 (11.5) | 39 (63.9) | 15 (24.6) | 0.359 |
|  | Group 2  n=25 | 5 (20) | 12 (48) | 8 (32) |  |
| Month 6 | Group 1  n=32 | 7 (21.9) | 20 (62.5) | 5 (15.6) | 0.361 |
|  | Group 2  n=15 | 2 (13.3) | 8 (53.3) | 5 (33.3) |  |
| Month 9 | Group 1  n=25 | 5 (20) | 12 (48) | 8 (32) | 0.775 |
|  | Group 2  n=14 | 3 (21.4) | 8 (57.1) | 3 (21.4) |  |
| Month 12 | Group 1  n=21 | 7 (33.3) | 8 (38.1) | 6 (28.6) | 0.211 |
|  | Group 2  n=7 | 0 (0) | 4 (57.1) | 3 (42.9) |  |
| Insomnia scale score | | | | | |
| Month 3 | Group 1  n=63 | 15 (23.8) | 34 (54) | 14 (22.2) | 0.213 |
|  | Group 2  n=26 | 2 (7.7) | 17 (65.4) | 7 (26.9) |  |
| Month 6 | Group 1  n=33 | 6 (18.2) | 19 (57.6) | 8 (24.2) | 0.110 |
|  | Group 2  n=15 | 0 (0) | 8 (53.3) | 7 (46.7) |  |
| Month 9 | Group 1  n=25 | 5 (20) | 14 (56) | 6 (24) | 0.888 |
|  | Group 2  n=14 | 2 (14.3) | 8 (57.1) | 4 (28.6) |  |
| Month 12 | Group 1  n=21 | 3 (14.3) | 14 (66.7) | 4 (19) | 0.519 |
|  | Group 2  n=7 | 2 (28.6) | 3 (42.9) | 2 (28.6) |  |
| Appetite loss scale score | | | | | |
| Month 3 | Group 1  n=63 | 5 (7.9) | 40 (63.5) | 18 (28.6) | 0.496 |
|  | Group 2  n=26 | 3 (11.5) | 13 (50) | 10 (38.5) |  |
| Month 6 | Group 1  n=33 | 4 (12.1) | 20 (60.6) | 9 (27.3) | 0.837 |
|  | Group 2  n=15 | 1 (6.7) | 10 (66.7) | 4 (26.7) |  |
| Month 9 | Group 1  n=26 | 1 (3.8) | 20 (76.9) | 5 (19.2) | 0.066 |
|  | Group 2  n=14 | 3 (21.4) | 6 (42.9) | 5 (35.7) |  |
| Month 12 | Group 1  n=22 | 3 (13.6) | 13 (59.1) | 6 (27.3) | 0.715 |
|  | Group 2  n=7 | 1 (14.3) | 3 (42.9) | 3 (42.9) |  |
| Constipation scale score | | | | | |
| Month 3 | Group 1  n=63 | 5 (7.9) | 46 (73) | 12 (19) | 0.208 |
|  | Group 2  n=26 | 4 (15.4) | 14 (53.8) | 8 (30.8) |  |
| Month 6 | Group 1  n=33 | 4 (12.1) | 21 (63.6) | 8 (24.2) | 0.398 |
|  | Group 2  n=15 | 4 (26.7) | 7 (46.7) | 4 (26.7) |  |
| Month 9 | Group 1  n=26 | 1 (3.8) | 16 (61.5) | 9 (34.6) | 0.027 |
|  | Group 2  n=14 | 4 (28.6) | 9 (64.3) | 1 (7.1) |  |
| Month 12 | Group 1  n=22 | 3 (13.6) | 12 (54.5) | 7 (31.8) | 0.653 |
|  | Group 2  n=7 | 1 (14.3) | 5 (71.4) | 1 (14.3) |  |
| Diarrhea scale score | | | | | |
| Month 3 | Group 1  n=63 | 6 (9.5) | 47 (74.6) | 10 (15.9) | 0.669 |
|  | Group 2  n=26 | 4 (15.4) | 19 (73.1) | 3 (11.5) |  |
| Month 6 | Group 1  n=33 | 5 (15.2) | 20 (60.6) | 8 (24.2) | 0.806 |
|  | Group 2  n=15 | 2 (13.3) | 8 (53.3) | 5 (33.3) |  |
| Month 9 | Group 1  n=25 | 3 (12) | 16 (64) | 6 (24) | 0.680 |
|  | Group 2  n=14 | 2 (14.3) | 7 (50) | 5 (35.7) |  |
| Month 12 | Group 1  n=22 | 2 (9.1) | 15 (68.2) | 5 (22.7) | 0.469 |
|  | Group 2  n=7 | 0 (0) | 4 (57.1) | 3 (42.9) |  |
| *Abbreviations. HAS: Haute Autorité de Santé; HCC: hepatocellular carcinoma.* | | | | | |

# Supplementary Table 5C: Global health score by analysis group: HCC cohort

|  | | **Improved** | **Stable** | **Deteriorated** | **P value** |
| --- | --- | --- | --- | --- | --- |
| Global health score, n (%) | | | | | |
| Month 3 | Group1  n=57 | 12 (21.1) | 30 (52.6) | 15 (26.3) | 0.982 |
|  | Group2  n=26 | 5 (19.2) | 14 (53.8) | 7 (26.9) |  |
| Month 6 | Group1  n=30 | 5 (16.7) | 10 (33.3) | 15 (50) | 0.057 |
|  | Group2  n=15 | 4 (26.7) | 9 (60) | 2 (13.3) |  |
| Month 9 | Group1  n=24 | 8 (33.3) | 9 (37.5) | 7 (29.2) | 0.661 |
|  | Group2  n=13 | 5 (38.5) | 3 (23.1) | 5 (38.5) |  |
| Month 12 | Group1  n=23 | 5 (21.7) | 12 (52.2) | 6 (26.1) | 0.798 |
|  | Group2  n=7 | 2 (26.6) | 4 (57.1) | 1 (14.3) |  |
| *Abbreviations. HAS: Haute Autorité de Santé; HCC: hepatocellular carcinoma.* | | | | | |
